# Multi‐omics integrative analysis identified SNP‐methylation‐mRNA: Interaction in peripheral blood mononuclear cells

**DOI:** 10.1111/jcmm.14315

**Published:** 2019-05-20

**Authors:** Yi‐Hua Lu, Bing‐Hua Wang, Fei Jiang, Xing‐Bo Mo, Long‐Fei Wu, Pei He, Xin Lu, Fei‐Yan Deng, Shu‐Feng Lei

**Affiliations:** ^1^ Center for Genetic Epidemiology and Genomics, School of Public Health Medical College of Soochow University Suzhou P. R. China; ^2^ Department of Epidemiology and Health Statistics, School of Public Health Nantong University Nantong P. R. China; ^3^ Jiangsu Key Laboratory of Preventive and Translational Medicine for Geriatric Diseases Soochow University Suzhou P. R. China

**Keywords:** Causal Inference Test, DNA methylation, integrative analysis, MeQTLs, multi‐omics

## Abstract

Genetic variants have potential influence on DNA methylation and thereby regulate mRNA expression. This study aimed to comprehensively reveal the relationships among SNP, methylation and mRNA, and identify methylation‐mediated regulation patterns in human peripheral blood mononuclear cells (PBMCs). Based on in‐house multi‐omics datasets from 43 Chinese Han female subjects, genome‐wide association trios were constructed by simultaneously testing the following three association pairs: SNP‐methylation, methylation‐mRNA and SNP‐mRNA. Causal inference test (CIT) was used to identify methylation‐mediated genetic effects on mRNA. A total of 64,184 significant cis‐methylation quantitative trait loci (meQTLs) were identified (FDR < 0.05). Among the 745 constructed trios, 464 trios formed SNP‐methylation‐mRNA regulation chains (CIT). Network analysis (Cytoscape 3.3.0) constructed multiple complex regulation networks among SNP, methylation and mRNA (eg a total of 43 SNPs simultaneously connected to cg22517527 and further to PRMT2, DIP2A and YBEY). The regulation chains were supported by the evidence from 4DGenome database, relevant to immune or inflammatory related diseases/traits, and overlapped with previous eQTLs from dbGaP and GTEx. The results provide new insights into the regulation patterns among SNP, DNA methylation and mRNA expression, especially for the methylation‐mediated effects, and also increase our understanding of functional mechanisms underlying the established associations.

## 1 INTRODUCTION

Previous genome‐wide association studies (GWASs) have identified a large number of SNPs associated with complex traits and diseases.^1^, ^2^, ^3^ A big challenge after GWAS is to explain the functions of the identified SNPs, and to illustrate the mechanisms underlying the associations. Notably, only 7% of the identified variations are located in protein‐coding regions,^4^, ^5^ and the majority of diseases‐associated variations are unlikely to affect the protein functions by changing the amino acid sequence. DNA methylation plays an important role in regulating expression of target gene. DNA methylation at promoter is dynamically linked to gene activity, and could directly influence the patterns of gene expression and cellular differentiation.^6^ Aberrant DNA methylation in cancer was associated with abnormally regulated expression of normal cellular genes.^7^ Recently, disrupted DNA methylation patterns were established as a contributor to metabolic syndrome,^8^, ^9^ schizophrenia^10^, ^11^ and inflammatory or autoimmune disorders.^12^, ^13^ The feature and distribution of DNA methylation have been studied in a variety of tissues/cells, and these genome‐wide maps of DNA methylation have revealed interesting features and provided important insights into its potential functions in genome regulation.^14^, ^15^ However, the functional mechanism underlying the variation in DNA methylation itself is still largely unknown. Previous studies have revealed that DNA methylation at specific loci can be influenced by sequence variations.^16^, ^17^, ^18^, ^19^ So far, how these genetic variations exert their effects was largely unknown, for example, it is unknown whether the SNPs exert their effects on DNA methylation and ultimately affects the gene expression.

Genome‐wide expression quantitative trait locus (eQTL) analysis is a well‐known method to explore genetic effect of SNP on gene expression. This approach has extensively been used to investigate the associations between SNP and other target phenotype (eg methylation level^11^, ^16^, ^17^, ^18^, ^19^) or between two phenotypes (eg DNA methylation and gene expression^15^). Methylation quantitative trait locus (meQTL) analysis is a kind of improved eQTL method that has been used to investigate the associations between SNPs and the methylation levels.^16^, ^17^, ^18^, ^19^ Another study has assessed the associations between DNA methylation and gene expression, that is, expression quantitative trait methylation (eQTMs).^15^ However, such QTLs studies were largely limited to testing one single pair of association only. Because of lack of multiple‐omics data from the same set of samples, the complex triangle relationship between SNP, DNA methylation and mRNA expression was undefined yet.

This study conducted multi‐omics integrative analyses as well as causal inference test (CIT)^20^ to reveal the complex connections among SNP, DNA methylation and mRNA expression, and identified DNA methylation‐mediated regulation effects in peripheral blood mononuclear cells (PBMCs), a commonly used target cell in immunity studies.

## 2 MATERIALS AND METHODS

### 2.1 Subjects and PBMC isolation

The study was approved by the Institutional Research Ethic Board at Soochow University. Table S1 showed the basic characteristics of 43 study subjects. The human subjects included 43 unrelated Chinese Han adult females from Suzhou city of China, which were recruited originally for identifying risk molecules of rheumatoid arthritis. Subjects were excluded from serious diseases involving vital organs (brain, liver, kidney, heart or lung). All subjects signed informed consent forms before entering this project. A total of 15 ml peripheral blood was collected and stored in sodium‐citrate‐supplemented vacuum tubes. PBMCs were isolated using density gradient centrifugation using Lymphoprep (Sigma, life science, USA). PBMCs were divided into two equal parts, one for DNA extraction, and the other for RNA extraction after treatment of Trizol reagent (Invitrogen, Carlsbad, CA) to avoid RNA degradation.

### 2.2 Genome‐wide SNP genotyping, DNA methylation profiling and transcriptome profiling

DNA was extracted from the isolated PBMCs using phenol‐chloroform extraction and ultrapurification method.^21^ The quality of extracted DNA was first tested by 0.8% agarose gel electrophoresis to check the integrity (usually > 10KB main band). The OD260/280 of 1.7‐1.9 by NanoDrop ND‐1000 (Thermo Scientific, Wilmington, Delaware) spectrophotometer was the QC cutoff of the DNA purification. Affymetrix Genome‐Wide Human SNP Array 6.0 chips were used for SNP genotyping by following the protocol recommended by the manufacturer. The experiments were performed in the laboratory of CapitalBio Corporation (Beijing, China). We used the contrast QC greater than 0.4 for quality control. A total of 909,622 SNPs in each subject were genotyped. After excluded the SNPs with a minor allele frequency less than 5%, or a call‐rate less than 95%, 551,745 SNPs were finally used in further analysis. All analyses are based on human reference genome 37 version assembly annotations.

DNA methylation profiling was performed with Illumina 450K Infinium Methylation BeadChip according to the manufacturer's instructions in the laboratory of CapitalBio Corporation (Beijing, China). DNA methylation data quality control consist of sample QC (subjects with more than 5% probes with a detection *P* > 0.05 were removed) and probe QC (probes with a detection *P* > 0.05 more than 5% subject were excluded). DNA methylation data normalization contains background adjustment, colour‐bias adjustment, quantile normalization and beta mixture quantile (BMIQ) method normalization. The background adjustment was performed in GenomeStudio. Then, colour‐bias adjustment and quantile normalization were performed in the R package lumi, followed by the BMIQ normalization to eliminate the bias between probe types. The methylation level was measured as beta (β) = M/(M + U), in which M was the methylated signals and U was the unmethylated signals. The β value ranges continuously from 0 (unmethylated) to 1 (fully methylated). After normalization, 416 285 methylated sites were left for further data processing.

Total RNA was extracted from PBMCs according to the instructions recommended by the manufacturer. The OD260/280 of ≥1.8 was the QC cutoff of the RNA purification (NanoDrop ND‐1000 spectrophotometer). RNA integrity was determined with 1% formaldehyde denaturing gel electrophoresis and Agilent 2100 Bioanalyzer. 28S/18S rRNA ratio of ≥1.5 and RIN >7 were used as the eligible criteria. The transcriptome‐wide mRNA expression was profiled using lncRNA&mRNA Human Gene Expression Microarray V4.0 (CaptialBio Corp, Beijing, China). The data were extracted by Agilent Feature Extraction (V10.7). The data summary, normalization and quality control were performed with GeneSpring GX program (V12.0). The log2 transformation was applied using the Adjust Data function. The probes with less than 80% of detection rate and/or incomplete annotation information were filtered (Multiexperiment Viewer (MeV) software (http://www.tm4.org)). Subsequently, a total of 17,566 unique mRNA probes were used for further analysis.

### 2.3 Quantitative trait locus analyses for three pairs (SNP & methylation, SNP & mRNA and methylation & mRNA

All the three quantitative trait locus (QTL) analyses were performed with the MatrixEQTL package modelled in R software (freely available at http://cran.r-project.org/).^15^, ^22^, ^23^, ^24^, ^25^ Here, we defined the QTL analyses for three association pairs (SNP & methylation, SNP & mRNA and methylation & mRNA) as meQTL, eQTL and eQTM, respectively. Multivariate linear regression analysis was conducted for each QTL analysis after adjusting for age and disease states of rheumatoid arthritis. Compared to trans‐effects, cis‐effects were much larger and be more stable.^26^, ^27^, ^28^, ^29^, ^30^ So, in order to enhance the reliability of the results, this study focused on the cis‐effect. SNPs located within 1 megabase (Mb) on either side of methylation sites was supposed to exert local effect (cis‐meQTLs). For the cis‐eQTL analysis, SNPs were also confined within 1 Mb distant from the transcription start site (TSS) or transcription end site (TES) of mRNAs. For the cis‐eQTM analysis, methylation sites were confined within 1 Mb distant from TSS or TES of mRNAs. Benjamini‐Hochberg false‐discovery rate (FDR) was used to correct for multiple testing.

### 2.4 CIT for DNA methylation‐mediated genetic effect on mRNA expression

To identify DNA methylation‐mediated effect on mRNA expression, we first constructed the associated trios according to the analysis results of the above three pairs. The associated trios were generated according to the physical positions of SNPs and methylation sites in genes with known official names. Herein, CIT^31^, ^32^ was applied to identify methylation‐mediated association between SNPs and mRNAs. Briefly, the causal inference simultaneously requires the following four criteria: SNP and mRNA expression is associated; SNP is associated with methylation level after adjusting for mRNA; Methylation is associated with mRNA after adjusting for SNP; and SNP is independent of mRNA expression after adjusting for methylation level.^31^, ^32^ The covariates, age and disease status, were adjusted in the above four tests. The maximum of the test *P*‐values was reported as the CIT *P*‐Value. CIT was performed in SAS 9.2 software (SAS Institute Inc, Cary, North Carolina).

### 2.5 Construction of SNP‐methylation‐mRNA interaction network

Based on CITs, the fulfilled association trios (SNP‐methylation‐mRNA) were selected to construct gene regulatory network. All network data were visualized using open source bioinformatics software Cytoscape 3.3.0 (Institute of Systems Biology in Seattle).^33^

### 2.6 Linkage disequilibrium analysis

Since multiple nearby SNPs from SNP‐methylation‐mRNA regulatory chains were simultaneously connected to single methylation site, we conducted linkage disequilibrium (LD) analysis for those nearby SNPs in HaploView 4.2 using the data of 1000 Genomes Project^34^ (2504 volunteer donors from various ethnic populations). We also obtained the LD measure *r* ^2^, which represents the statistical correlation between two SNPs of interest and is frequently used in LD mapping because of its statistical advantages and strong theoretical basis for population genetics. When *r* ^2^ = 0, it shows that the two loci are completely independent, and *r* ^2^ = 1 means SNPs at the two loci sharing the same frequency.

### 2.7 The identified SNP‐methylation‐mRNA chains overlapped with previous results

To find whether the identified associations in significant SNP‐methylation‐mRNA chains were reported by previous studies, we searched the databases of Phenotype‐Genotype Integrator (PheGenI) (www.ncbi.nlm.nih.gov/gap/PheGenI/), National Human Genome Research Institute (NHGRI) Catalog of published GWAS (GWAS Catalog), GTEx Portal (https://www.gtexportal.org/), and the 4DGenome, a general repository for chromatin interaction data (https://4dgenome.research.chop.edu/).

## 3 RESULTS

### 3.1 Cis‐meQTL identification and distribution characteristics

**Figure 1:**
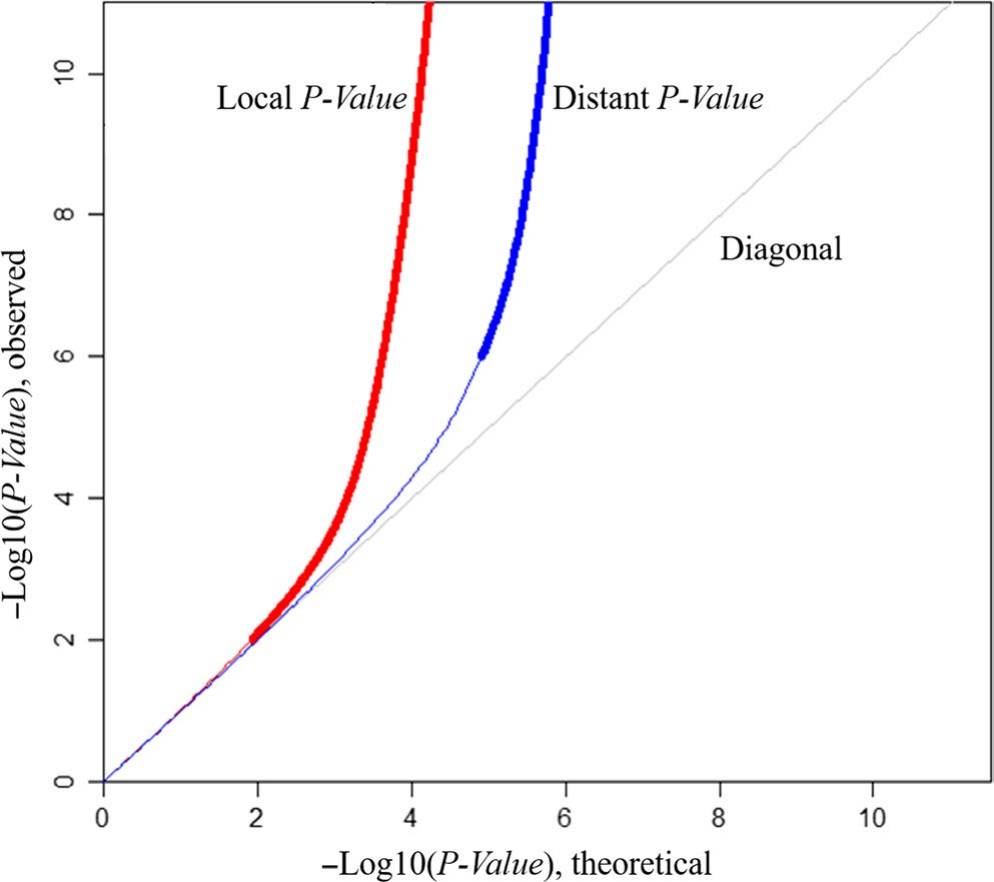
Quantile‐quantile plots of the associations from meQTL analyses. Local *P‐*value: *P‐*value from cis‐meQTLs, in which the SNPs located within 1 megabase (Mb) on either side of methylation sites; Distant *P‐*value: *P‐*value from trans‐meQTLs, in which the SNPs located outside 1 megabase (Mb) on either side of methylation sites

A total of 551 745 genotyped SNPs and 416 285 methylation levels were tested. Among the 144 470 159 tested cis‐meQTL pairs, 64 184 pairs were significant (FDR < 0.05), which corresponded to 40 896 unique SNPs and 16 033 unique methylation sites (Table 1 and Table S2). As shown in Figure 1, the plots significantly deviated from the reference line for both cis‐meQTLs and trans‐meQTLs, but more rapidly for cis‐meQTLs, suggesting that the significant regulation effects of SNPs on methylation were relatively stronger for cis‐meQTLs than for trans‐meQTLs in general.

**Table 1:** Summary of associations in meQTLs, eQTMs and eQTLs

	meQTLs	eQTMs	eQTLs
Test	SNP & methylation	Methylation & expression	SNP & expression
Window size	1 MB	1 MB	1 MB
FDR	5%	5%	5%
Number of tests	144 470 159	10 944 256	5 880 162
Maximum *P‐*value	2.22E‐05	1.28E‐05	4.39E‐06
Cis‐effect pairs	64 184	2 795	525
SNP	40 896	—	471
Methylation	16 033	2 090	—
mRNA	—	837	140

eQTLs, Expression quantitative trait loci, the association between SNP and gene expression; eQTMs, Expression quantitative trait methylation, the associations between DNA methylation and gene expression; FDR, Benjamini‐Hochberg false‐discovery rate; meQTLs, Methylation quantitative trait loci, the association between SNPs and methylation level.

**Figure 2:**
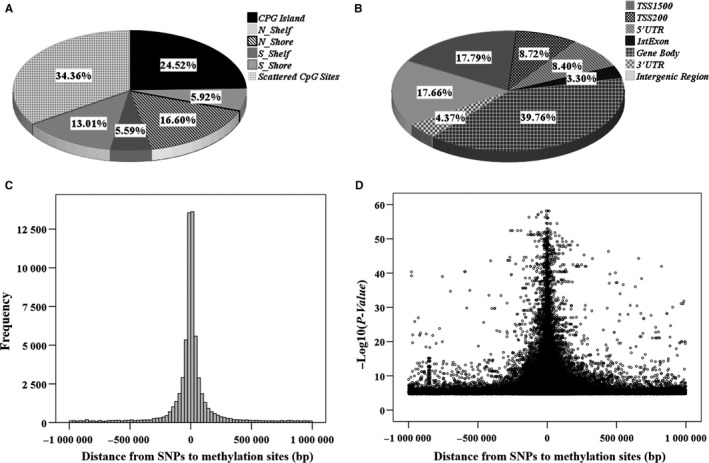
The distribution of cis‐meQTL associated methylation sites. (A) The distribution according to their positions in the UCSC CpG island region. (B) The distribution according to gene region feature category (UCSC). (C) The frequency distribution Note: Distance indicates the physical distance from SNP to their associated methylation site. (D) The distribution according to association significance (‐log10(*P‐*value)) against the physical distance from SNP to their associated methylation site

Specifically, for the methylation sites in CpG regions, we identified 42 131 (65.64%: 42 131/64 184) significant cis‐meQTL pairs. These corresponding methylation sites were distributed at CpG island (N = 15 738, 24.52%), CpG shore (N_shore and S_shore, 0‐2 Kb from CpG island) (N = 19 005, 29.61%) and CpG shelf (N_shelf and S_shelf, 2‐4 Kb from CpG island) (N = 7 388, 11.51%) (Figure 2A). The shores and shelves were annotated according to their chromosome orientation from the p‐ to q‐arms as in N‐ and S‐shores, respectively. As presented in Figure 2B, according to annotation of gene region feature category by UCSC (http://www.genome.ucsc.edu), the corresponding SNPs were distributed in gene body (39.76%), TSS 1500 (−1500 to −200 bp to TSS) region (17.79%), TSS 200 (−200 bp to TSS) region (8.72%), 5′‐UTR (8.40%), as well as 1st exons (3.30%).

The distribution of the physical distance from SNPs to their associated methylation sites for all the significant cis‐meQTLs (FDR < 0.05) was presented in Figure 2C. An obvious peak in frequency around the methylation sites was observed, suggesting that the SNPs from the significant meQTLs were enriched around methylation sites. Similarly, the most effective SNPs were enriched around the methylation sites, indicating that the closer to methylation sites, the greater effects of the SNPs on methylations in general (Figure 2D).

### 3.2 Identification of the SNP‐methylation‐mRNA regulation chain

We further tested the associations of the following two pairs, methylation‐mRNA by eQTMs and SNP‐mRNA by eQTLs. As shown in Table 1, we identified 2,795 local significant methylation‐mRNA associations (cis‐eQTMs) (FDR < 0.05), and 525 significant local effect SNP‐mRNA pairs (cis‐eQTLs) (FDR < 0.05). According to the physical positions of SNPs and methylation sites in the corresponding genes with official names, the significant pairs from the above three association tests were matched and the overlapped pairs were selected to construct SNP‐methylation‐mRNA association trios. Consequently, 745 trios were generated, which corresponded to 272 unique SNPs, 65 unique methylations and 47 unique target mRNAs. In‐depth CIT analysis showed that 464 trios fulfilled significant causal inference. Specifically, 191 SNPs were associated with mRNA expressions of 37 genes, which were dependently through 56 methylation sites. Notably, 21 SNPs were simultaneously associated with the same methylation site (cg22517527) and the same gene expression (PRMT2). The statistical results for the 464 methylation‐mediated genetic effects on mRNA expressions were detailed in Table S3, including effect directions and *P*‐values under all the tested conditions.

### 3.3 Construction of SNP‐methylation‐mRNA interaction network

**Figure 3:**
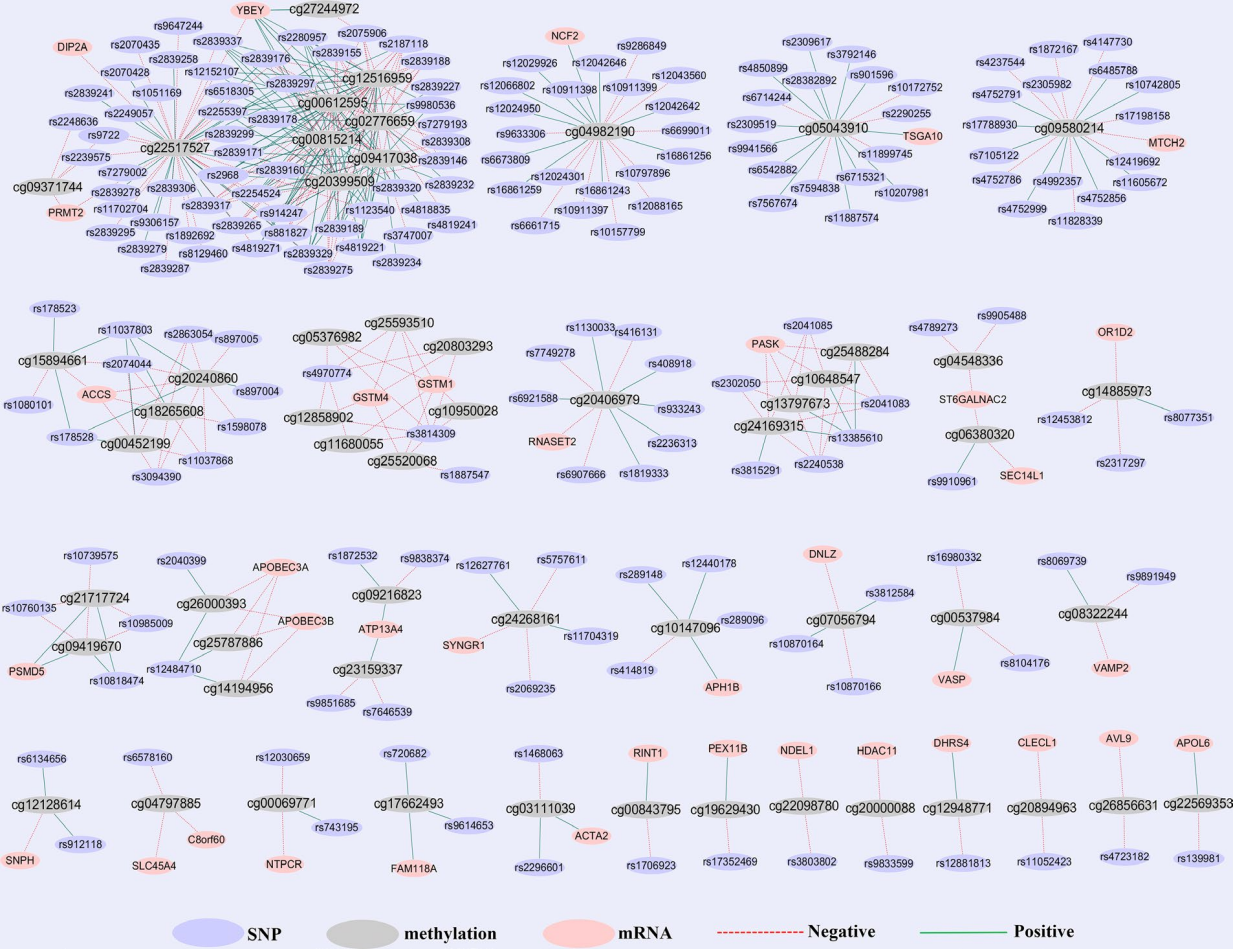
The constructed networks based on the significant SNP‐methylation‐mRNA regulatory chains. Cytoscape 3.3.0 was used to establish the networks. The SNP, DNA methylation and mRNA from significant CIT trios were imported. Purple nodes represent SNPs, grey nodes represent DNA methylation and pink nodes represent mRNA. Red dot edges represent negative regulation between two nodes. Green solid lines represent positive regulation between two nodes

Figure 3 demonstrated the epi‐genetic regulation patterns among 171 SNPs, 56 methylation sites and 37 target genes from 464 causal trios. We found a primary network and several small separate networks, which showed their complex regulation patterns. Notably, it was common that multiple SNPs are connected to a few limited methylation sites or mRNAs. For example, 43 SNPs simultaneously influenced methylation at cg22517527 and further regulated mRNA expression of PRMT2 (protein arginine methyltransferase 2), DIP2A (disco‐interacting protein 2 homolog A) and YBEY (ybeY metallopeptidase). Besides, 20 SNPs regulated YBEY expression through methylation at the cg20399509, and 30 SNPs regulated YBEY expression by methylation at the cg12516959.

### 3.4 LD analysis

**Figure 4:**
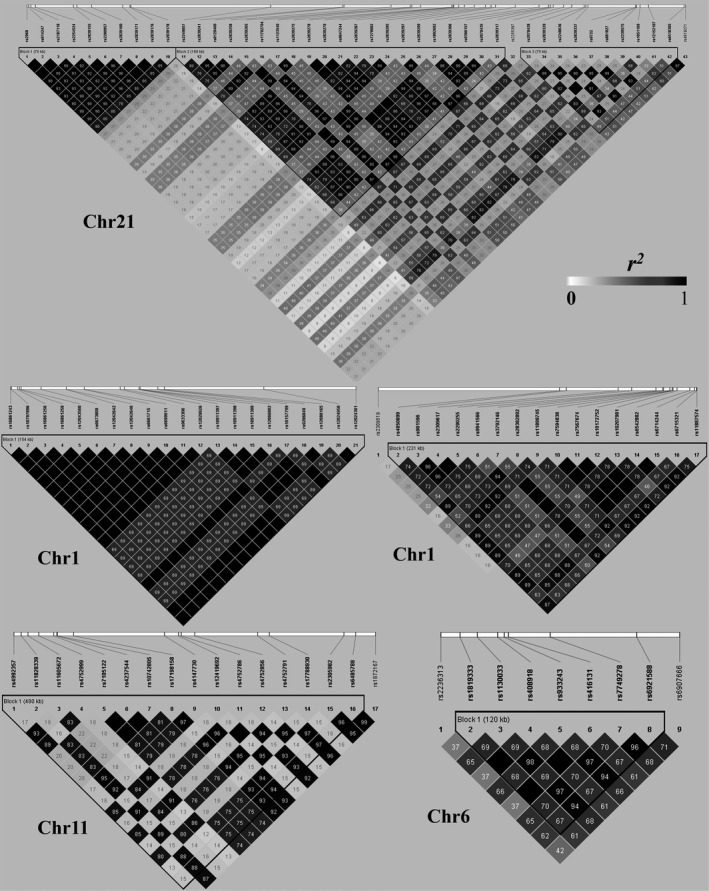
Linkage disequilibrium (LD) analysis. HaploView 4.2 was used to analyse the linkage disequilibrium. The SNPs used in LD analysis are from the significant SNP‐methylation‐mRNA regulatory chains. The shades of colour represent *r* ^2^, deeper colour represent the higher value of *r* ^2^. Each number in cell represents *r* ^2^ between neighbouring SNPs. The black cells without numbers means *r* ^2^ = 1

LD analyses for the SNPs physically located closely in the same chromosome were performed by using the HaploView and the genotype data of 1000 Genomes Project. As we expected, strong LD structure was detected for most of the analysed SNPs. As shown in Figure 4, we have found 7 LD blocks (3 in Chr21, 2 in Chr1, 1 in Chr11 and 1 in Chr6). For example, an SNP group (10 SNPs) in the Block 1 of Chr21 formed an extremely strong LD block (the first sub‐network in Figure 3), which simultaneously influenced five methylation sites (cg00612595, cg02776659, cg09417038, cg12516959 and cg20399509) and further regulated mRNA expression of YBEY. In the Block 1 of Chr1, 21 SNPs also formed a strong LD block, which correspond to the 21 SNPs‐cg04982190‐NCF2 regulation chains (the second sub‐network in Figure 3). Besides, another group (16 SNPs) had strong LD in chromosome 1, which were simultaneously connected to cg05043910 and further regulated mRNA expression of TSGA10 (the third sub‐network in Figure 3).

### 3.5 Supported evidence from 4DGenome database for the identified SNP‐methylation‐mRNA chains

To find additional evidence to support the identified SNP‐methylation‐mRNA chains, we searched the 4DGenome database based on their physical positions (hg19). The information of the SNP‐Methylation‐mRNA chains, their located interactions, detection methods and the detected cells/tissues were listed in Table S4. We found 89 significant interactions that correspond to 38 unique SNP‐Methylation‐mRNA chains in 23 types of cells/tissues including CD4 T cell, an important immunity related cell. Especially for the rs8069739‐cg08322244‐VAMP2, about 33 interactions were identified in a variety of cells, for example, in the CD4 T cells, rs8069739 and VAMP2 were located in Interactor B and cg08322244 was located in Interactor A.

### 3.6 The SNP‐methylation‐mRNA chains relevant to immune or inflammatory related diseases/traits

Among the 191 SNPs and 37 genes involved in the identified causal trios, 19 SNPs and 7 genes were reported to be significantly associated with human diseases/traits (Table S5), as archived in the NHGRI database and the database of Genotypes and Phenotypes (dbGaP). The associated diseases/traits included rheumatoid arthritis, multiple sclerosis, systemic lupus erythematosus, inflammatory bowel disease and crohn disease, which are immune or inflammatory related diseases/traits.

### 3.7 The identified SNP‐methylation‐mRNA chains overlapped with previous eQTLs from dbGaP and GTEx

The identified regulation chains also probably provide functional explanations for previous eQTL results. After searched the dbGaP according to SNP ID, 16 eQTLs had been reported in lymphoblastoid and liver, which correspond to 13 unique SNPs and 6 genes. Meanwhile, the GTEx Portal was also retrieved for more supportive information, and it was found 112 eQTLs results containing 74 unique SNPs and 10 genes (Table S6).

## 4 DISCUSSION

DNA methylation is known as an important epigenetic regulatory factor in mediating the correlations between genetic variants and mRNAs.^35^, ^36^ EQTL analysis is a typical and powerful statistical method in explaining the functional link between SNP and disease/trait.^27^, ^37^, ^38^, ^39^ Several studies have supported that cis‐acting QTLs have large effect sizes that can be detected in a relatively small sample less than 100 subjects.^16^, ^40^, ^41^, ^42^ A large number of cis‐meQTL associations identified in our study have suggested that DNA methylation levels were under strong genetic influence. Previous meQTL studies also found abundant local effects in other tissues.^22^, ^43^, ^44^, ^45^ Furthermore, an obvious peak observed around the methylation sites suggested that the physical distance seemed to have large effect on their associations, that is, the more close to methylation site, the greater effect for the associations. Our findings suggested that methylation sites were typically associated with SNPs in close proximity. The significant SNPs in our cis‐meQTL analysis were mostly located in gene regions, including gene body and the promoter. It is because that genetic influence on the human methylome involves heterogeneous processes and is predominantly dependent on local sequence context at the meQTL sites.^45^, ^46^

This study represents the first efforts of conducting integrative multiple omics analyses by multiple QTL tests and in‐depth CIT to more comprehensively reveal methylation‐mediated regulation effects. CIT provides a highly interpretable quantitative measure for a trio of variables when association between two implies causation and the third is a potential mediator.^32^ This method is theoretically and computationally accessible in disentangling molecular relationships,^47^ and was proposed as a novel statistical framework in which existing notions of causal mediation were formalized into a hypothesis test.

The identified 464 regulation chain of SNP‐methylation‐mRNA suggest that DNA methylation can emerges both as marker and determinant.^15^ For instance, we found 43 SNPs further regulates gene expression of PRMT2, DIP2A and YBEY by regulating methylation levels of cg22517527. Two SNPs (rs4970774 and rs3814309) negatively regulated the GSTM1 and GSTM4 by negatively controlling seven methylated sites collectively. The SNP rs12484710 positively regulated cg26000393, cg25787886 and cg14194956 so that to influence the expression of APOBEC3A and APOBEC3B. LD analysis was conducted in SNPs physically located closely by using the HaploView and 1000 Genomes Project. Strong LD structure was identified for most of the analysed SNPs. Nevertheless, the SNPs closest to the significant methylation site is likely to be most effective in regulation, but when multiple SNPs are in strong LD in the detected region, it is challenging to discriminate which SNP within the same LD region is truly causative. Moreover, whether and how the SNPs influences the methylation structure and then regulates gene expression is unclear yet. These regulatory networks can provide a basis for future functional studies. Herein, we propose that some DNA sequence variants, through changing the methylation levels, hence influences the gene expressions.

To find more evidence to support the identified SNP‐methylation‐mRNA regulatory chains, we searched the 4DGenome. 4DGenome is a public database that archives and disseminates chromatin interaction data. It covers all major experimental technologies including chromosome conformation capture (3C), chromosome conformation capture‐on‐chip (4C), chromosome conformation capture carbon copy, ChIA‐PET, Hi‐C, Capture‐C and a computational prediction of IM‐PET for detecting chromatin interactions. The interaction paired sequence tags archived in this database implies the corresponding pair of DNA regions is in close physical proximity and probably functionally interacts. We found 89 results including 38 unique SNP‐methylation‐mRNA chains overlapped with the 4DGenome database. The results from 4DGenome database provide additional understanding of chromatin architecture how SNP, methylation and mRNA are tightly related.

The identified regulation chains probably provide functional explanations for the associations of SNPs and diseases. We searched for the Phenotype‐Genotype Integrator and GWAS Catalog. PBMCs consist of several important immunity cells, which play a decisive role in the process of immune or inflammatory response. It was found some SNPs and genes in SNP‐methylation‐mRNA chains were related to the immune or inflammatory disease/trait by searching for the database. For instance, RNASET2 regulated by rs1819333 is associated with inflammatory bowel diseases and crohn disease, which further illustrated that significant association of SNPs and genes in SNP‐methylation‐mRNA chains, may play a decisive role in the process of immune response in PBMCs. These overlapped results suggested that the methylation is a probable functional mechanism in connecting SNP with the susceptibility to diseases. We further validated the associations between SNPs and mRNAs in SNP‐methylation‐mRNA chains in dbGAP and GTEx Portal, and found 16 overlap results in dbGAP and 112 overlap results in GTEx Portal.

This study had several potential limitations. First, the relatively small sample size may offer limited power in detecting minor‐ or modest‐effect meQTLs/eQTMs/eQTLs. Second, the inferred interaction patterns were based on multi‐omics data, further cellular and molecular experiments will be helpful to validate the findings. Third, it was probably inappropriate to extend the PBMC expression regulatory pattern to other cells or tissues because of the high tissue‐ or cell‐specificity as mentioned above. Fourth, the subjects concerning only women may limit the extensions of the results in male or mix sample (both male and female) because gender‐specific genetic architecture is common in humans. Last, the cross‐reactive probes and polymorphic CpGs in Illumina 450K Infinium Methylation BeadChip probably have confusing impacts on methylation readouts*.* 48, 49

In summary, our study comprehensively investigated the (epi‐) genetic architecture underlying the variation of methylation expression, and illustrated SNP‐methylation‐mRNA regulation pattern by in‐depth CIT analysis in human PBMCs by using multi‐omics integrative strategy. The results provide new insights into the regulation patterns among SNP, DNA methylation and mRNA expression, especially for the methylation‐mediated effects, and also increase our understanding of functional mechanisms underlying the established associations. The results would further facilitate the investigations of PBMC‐related immune physiological process and immunological diseases in the future.

## DATA SHARING STATEMENT

The microarray data for methylation have been submitted to the GEO database with accession number GSE111942.

## CONFLICT OF INTEREST

The authors declared that we have no conflict of interest to this work.

## AUTHOR CONTRIBUTION

Study conception and design: Shu‐Feng Lei, Fei‐Yan Deng and Yi‐Hua Lu; Acquisition of data: Long‐Fei Wu, Fei Jiang, Pei He and Xin Lu; Analysis and interpretation of data: Yi‐Hua Lu, Bing‐Hua wang, and Xing‐Bo Mo; Drafting of manuscript: Yi‐Hua Lu; Critical revision: Fei‐Yan Deng and Shu‐Feng Lei. All authors have participated in revising the manuscript critically and gave their final approval of the version to be submitted.
